# Medical Words

**Published:** 1891-10

**Authors:** 


### MEDICAL WORDS.

The *Medical News* initiates a reform in the spelling of certain words, which we think, on the whole, very commendable. The medical editorial staff of the *News* hereafter will use the simple *e* for the older form, *æ* and *œ*. Thus: Hemorrhage, Anesthesia, Etiology, Esophagus, Cecum, Gonorrhea, etc., etc. It is not yet brave enough, however, to change Cæsarean, Nævus, Amœba, Pharmacopœia, etc.

Speaking of orthography suggests etymology, and here we wish to raise our voice in protest against a word which has, of late years, crept into the language. Whatever else may be said of *electrocution*, the word itself is all wrong, just as *vulvitis*, *ovaritis* and *antifebrin* are wrong. These are hybrids, bastards, deformed and misshaped. Rockwell proposes *electro-execution*, which is not very much of an improvement, notwithstanding the hyphen. *Electrothanasia* is suggested by Peterson, and this seems to answer the requirements of etymology and euphony fairly well. But that it will ever be pronounced "trippingly on the tongue" we seriously doubt.

